# Examining State Policies and Administrative Factors as Determinants of Consumer-Reported Unmet Service Needs in Publicly Funded Home- and Community-Based Services in the United States

**DOI:** 10.3390/jmahp13040051

**Published:** 2025-10-02

**Authors:** Romil R. Parikh, Tetyana P. Shippee, Benjamin Langworthy, Zheng Wang, Stephanie Giordano, Eric Jutkowitz

**Affiliations:** 1 Division of Health Policy & Management, University of Minnesota School of Public Health, Minneapolis, MN 55455, USA; 2 Biostatistical Design and Analysis Center, Clinical and Translational Science Institute, University of Minnesota, Minneapolis, MN 55455, USA; 3 Department of Biostatistics and Research Decision Sciences, Merck & Co., Inc., Rahway, NJ 07065, USA; 4 Human Services Research Institute, Cambridge, MA 02140, USA; 5 Department of Health Services, Policy and Practice, Brown University School of Public Health, Providence, RI 02903, USA; 6 Center of Innovation in Long Term Services and Supports, Providence VA Medical Center, Providence, RI 02908, USA; 7 Evidence Synthesis Program Center, Providence VA Medical Center, Providence, RI 02908, USA

**Keywords:** Medicaid, long-term services and supports, access, quality assurance, policy evaluation, home healthcare

## Abstract

Access to home- and community-based services (HCBSs) varies substantially between states. Yet, it is unknown how state-level policies and administrative factors impact consumer-reported unmet service needs, an important indicator of HCBS access and quality. Using the National Core Indicators—Aging and Disability Adult Consumer Survey (2016–2019; *n* = 13,654 community-dwelling older adults, 13 states), we examined associations between unmet HCBS needs with four state-level factors: HCBS spending relative to institutional care spending, HCBS spending per client, percentage of Medicaid beneficiaries in managed care, and Medicaid expansion; and funding program. In the adjusted logistic regression model, the odds of overall unmet HCBS needs were lower with higher percentage Medicaid beneficiaries in managed care (adjusted odds ratio [aOR], 0.92; 95% confidence interval [CI], 0.89–0.96) and Medicaid expansion (aOR, 0.80; 95% CI, 0.73–0.87) but greater with higher HCBS spending relative to institutional care spending (aOR, 1.19; 95% CI, 1.11–1.28). Compared to Medicaid waiver, odds of unmet HCBS needs were significantly lower among consumers in Managed Long-Term Services and Supports (aOR, 0.67; 95% CI, 0.61–0.74) and Program of All-Inclusive Care for the Elderly (PACE; aOR, 0.39; 95% CI, 0.31–0.49). State policies and administrative factors are important place-based determinants of HCBS consumers’ unmet HCBS needs/access; and warrant consideration in HCBS quality assurance and improvement.

## 1. Introduction

Medicaid is the primary payer for HCBS in the United States, with over 4.5 million beneficiaries receiving HCBS annually, including older adults and people with disabilities who rely on these services to remain in community settings [1]. Since 2013, states have continually expanded home and community-based services (HCBS) through section 1915 waivers and federally funded initiatives, each year spending more on HCBS than on nursing home care [1]. Most people prefer receiving long-term services and support (LTSS) in their homes or community settings over nursing homes [2,3]. This consumer preference has led to a significant reallocation of public LTSS funds, with Medicaid spending more on HCBS than costlier nursing home care [4,5]. However, there are large variations in HCBS spending across states due to differences in factors such as cost of living and service coverage. For instance, in 2019, annual Medicaid HCBS spending per client in the United States ranged from a low of USD 126.87 in Florida to a high of USD 867.32 in New York [6].

Prior research has primarily examined state variation in HCBS spending, availability of services, and use of managed care, with mixed evidence about their impact on system-level outcomes such as institutionalization rates, expenditures, and service coverage [3,7,8,9,10]. However, data on the association between state policies and consumer experiences of unmet HCBS needs (defined as situations where individuals require but do not receive adequate services) is lacking. This is a critical gap, as unmet HCBS needs are not only indicators of poor access and quality, but are also strongly linked to caregiver burden, avoidable hospitalizations, and premature nursing home placement [11,12,13]. The gap in the literature is twofold. First, prior research has predominately assessed system-level metrics (e.g., expenditures, service coverage, and healthcare utilization) rather than consumer-centered outcomes that directly reflect the adequacy of HCBS delivery [7,8,9,10]. Second, while unmet service needs have been studied at the individual level, little is known about how state-level policies and administrative structures influence these needs among HCBS consumers [14,15,16]. Given the growing emphasis on person-centered quality measures, understanding these policy-consumer linkages is essential for advancing equity, accountability, and quality improvement in HCBS [11].

This study addresses gaps mentioned above by using the National Core Indicators–Aging and Disability Adult Consumer Survey (NCI-AD), one of the few national data sources capturing consumer-reported HCBS experiences across states [14,15,16]. We examined associations between four state-level policies and consumer-reported needs among publicly funded HCBS users: (1) HCBS spending relative to institutional care spending, (2) HCBS spending per client, (3) the percentage of Medicaid beneficiaries enrolled in managed care, and (4) whether the state adopted Medicaid expansion. We selected these four state-level factors because each reflects a distinct policy-lever with potential implications for HCBS access and quality. HCBS spending relative to nursing home spending reflects the degree to which states prioritize community-based care over nursing facility care, a longstanding benchmark of “rebalancing” efforts [4,5]. HCBS spending per client reflects the depth of investment in each consumer’s services and thus may indicate whether resources are sufficient to meet individualized needs [6,7]. The percentage of Medicaid beneficiaries enrolled in managed care represents the degree to which states rely on managed LTSS to coordinate care, with prior studies suggesting both potential efficiencies and risks of service restriction [17,18,19]. Medicaid expansion broadens eligibility for low-income adults, potentially increasing access to HCBS for populations who might otherwise experience unmet service needs. This can occur directly through service coverage expansion and indirectly by influencing market supply (for example, expanded consumer-base can incentivize service providers to initiate or expand services by increasing market potential). Together, these four factors capture both financing priorities and administrative structures that vary substantially across states and may function as place-based determinants of consumers’ experiences of unmet HCBS needs. Furthermore, HCBS can be administered through different care programs such as the Medicaid Aging and Disability waiver, Managed LTSS, Older American’s Act (OAA), and the Program of All-Inclusive Care for the Elderly (PACE), each of which have a different level of care coordination and administrative structure (summarized in Table 1). Each of these programs may impact access to HCBS; consequently, influencing consumers’ unmet service needs. Accordingly, we asked the following research question: What are the associations between these state-level policies and administrative factors and consumer-reported unmet HCBS needs after adjusting for variability within states?

## 2. Methods

Our study was reviewed and approved by the Institutional Review Board at the University of Minnesota.

### 2.1. Data Source and Survey Methods

We used data from the NCI-AD Adult Consumer Survey, which evaluates publicly funded LTSS through a standardized in-person assessment measuring quality of life, health outcomes, and service coordination, access, choice, and safety. This survey has been described in detail in previous publications [14,15,16]. The NCI-AD survey is unique in capturing consumers’ self-reported unmet service needs across specific service categories. The survey is conducted in partnership with ADvancing States, Human Services Research Institute (HSRI), and state Medicaid, aging, and disability agencies. State participation in the NCI-AD survey program is voluntary; however, for inclusion in data analytics and reporting, each state is required to have a minimum of 400 respondents. The sampling process for the survey is determined by each participating state. A probabilistic approach is used to draw participants from the population eligible for HCBS, typically within specific funding streams, with the goal of achieving no more than a 5% margin of error at the 95% confidence level. Prior to administering interviews, states compile detailed background information from administrative records, including demographics, legal and clinical status, and service use, which are then linked to the survey responses. This linkage creates a comprehensive profile that integrates administrative and survey data for each individual. The “Background Information” section of the instrument draws on sources such as Medicaid billing, case management systems, and managed care records to capture details on race, gender, disability status, Alzheimer’s disease and related dementias, and mental health conditions. Administrative data are especially important for verifying service types, most often through the Medicaid Management Information System, rather than relying exclusively on self-reports.

Sample selection generally takes place three to four months into the start of the survey cycle, though respondents may not be interviewed until as late as eight months afterward due to the extended fielding window. Demographic and service information in the NCI-AD is drawn primarily from administrative records, but if records are incomplete, respondents are asked these questions directly at the close of the survey. Certain elements such as primary LTSS funding source, program enrollment, services received, service duration, use of self-direction, and guardianship status, must be obtained from state records. To be eligible for inclusion, individuals must be actively using at least one LTSS (e.g., personal care, homemaker support, transportation) at a frequency of two or more times per week for approximately three months. Active service use is therefore a prerequisite for participation.

### 2.2. Study Population

**Figure 1:**
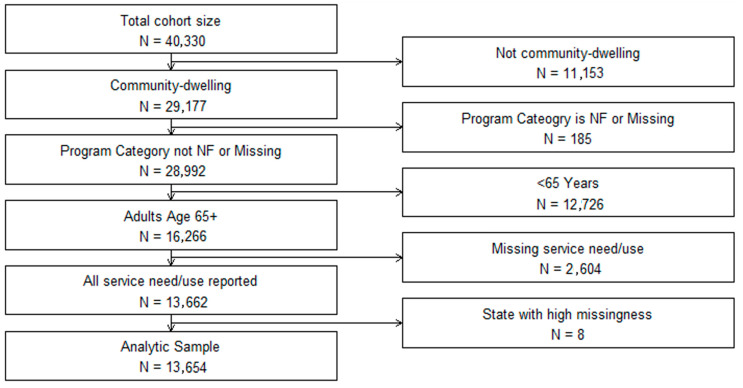
Participant Selection Flow Diagram: The National Core Indicators—Aging and Disability Survey (2016–2019).

We merged data from three consecutive waves of the NCI-AD surveys (2016–2019, *n* = 40,330 respondents) from 13 states (CO, IN, KS, MN, MS, NE, NJ, NV, OR, TN, TX, VT, and WI; Figure 1). Sensitivity analyses comparing characteristics from individual waves to those from the combined sample did not indicate any meaningful differences in participant characteristics. After excluding those in institutional settings or <65 years of age, those in nursing homes or with missing data on waiver status and unmet service needs, and those from one state with excessive missing data (*n* = 8 respondents, with >75% missing data), our final analytic sample included 13,654 respondents who were community-dwelling older adults of age 65 years or more.

### 2.3. Dependent Variable: Unmet HCBS Needs

The primary dependent variable was consumer-reported unmet service needs in any HCBS. We also examined unmet service needs in six specific HCBS types: (1) personal care, (2) homemaker/chore services, (3) meal delivery, (4) adult day services, (5) transportation, and (6) caregiver support. The Background Information (BI) section in the survey recorded actual service use through the question, “What type of paid long-term care supports is the person receiving?” (Supplemental Table S1). In a subsequent question, respondents were asked, “Do the long-term care services you receive meet your current needs and goals?” Respondents replying “No” or “Some” were categorized as having unmet HCBS needs. A follow-up question asked which additional services would help meet their needs, allowing multiple selections: “What additional long-term care services might help you meet your needs and goals?” (Supplemental Table S1). Responses to this question helped identify unmet service needs for the six specific HCBS types mentioned above.

### 2.4. Independent Variable: State-Level Factors

Our primary independent variables were four state-level factors, established as important by prior literature [7,8,9,10], and obtained from the Kaiser Family Foundation public website (https://www.kff.org (accessed on 18 July 2023)): (1) Ratio of HCBS spending to institutional care spending, (2) HCBS spending per client (in USD 10K units), (3) percentage of Medicaid beneficiaries in managed care, and (4) Medicaid expansion under the Affordable Care Act (yes, no). Medicaid managed care refers to the delivery system in which states contract with managed care organizations (MCOs) usually on a capitated, per-member-per-month payment basis to coordinate and provide comprehensive Medicaid services to enrollees. We linked Kaiser data to the NCI-AD survey data using state identifiers. Each variable was calculated using data from the fiscal year preceding the survey wave.

### 2.5. Independent Variable: Consumer-Level Factors

Consumer-level covariates were selected based on prior literature [13,14,15,16]. These were obtained from the BI section based primarily on administrative records data. We obtained demographic data such as respondent’s race (Black, White, Hispanic, or other), sex (male/female), residence ZIP code classification (metropolitan, micropolitan, small town, rural), living arrangement (living alone or not), and (5) marital status (single, divorced, widowed). We obtained other covariates as potential confounders such as survey year, state identifier, funding program category (e.g., Managed LTSS, PACE, OAA, and Medicaid Aging & Disability waiver), insurance type, and the presence of a legal guardian for the respondent. Additional health-related consumer-level covariates obtained were overall self-rated health (5-point Likert scale from poor to excellent), and diagnosis of dementia, developmental disability, physical disability, brain injury, and/or mental illness, as documented in the BI section or during survey administration.

### 2.6. Statistical Analyses

We calculated descriptive characteristics of the study sample as frequencies and percentages for categorical variables and as medians (interquartile interval) for continuous variables. To evaluate associations between state-level factors and unmet HCBS needs, we constructed two sets of regression models and calculated odds ratios (OR) with 95% confidence intervals (CI). We conducted logistic regression with the four state-level independent variables, and all consumer-level covariates, state, and survey year. Next, we built generalized estimating equation (GEE) models including consumer-level covariates mentioned above, and accounting for respondent clustering by state-survey year. We considered one cluster for one state in one survey year (for example, Minnesota in 2018 would be considered as one cluster). All analyses were performed using R version 4.4.0 (R Core Team, 2024). We used *p* < 0.05 as the threshold for statistical significance.

## 3. Results

In our study sample of 13,654 respondents (Table 2), the median age was 77 years (interquartile interval, 71–84 years). Most participants were female (72%), white (60%), and had a physical disability (58%), had Medicare (91%), and lived in metropolitan areas (72%); and 13% had proxy respondents.

In our primary analysis (Table 3), using fixed-effects model, after adjusting for consumer-level covariates, the odds of reporting unmet HCBS needs were significantly lower among consumers in states with higher percentage managed care population (OR, 0.92, 95% CI, 0.89, 0.99, *p* < 0.001) and with Medicaid expansion (OR, 0.80, 95% CI, 0.73, 0.87, *p* < 0.001). The odds of reporting unmet HCBS needs were significantly greater among consumers in states with higher HCBS spending relative to institutional care (OR, 1.19, 95% CI, 1.11, 1.28, *p* < 0.001). HCBS spending per client showed no statistically significant relationship with unmet HCBS needs. Compared to Medicaid waiver program, the odds of unmet HCBS needs were significantly lower among consumers in Managed LTSS (OR, 0.67; 95% CI, 0.61, 0.74; *p* < 0.001) and PACE (OR, 0.39; 95% CI, 0.31, 0.49; *p* < 0.001). In regard to consumer characteristics, the odds of unmet HCBS needs were significantly lower among consumers with better self-rated health and those residing in rural areas compared to metropolitan areas (Table 3). The odds of unmet HCBS needs were significantly greater among consumers who were separated/ divorced (versus married) and those living with dementia, physical disability, or serious mental illness (Table 3).

**Figure 2:**
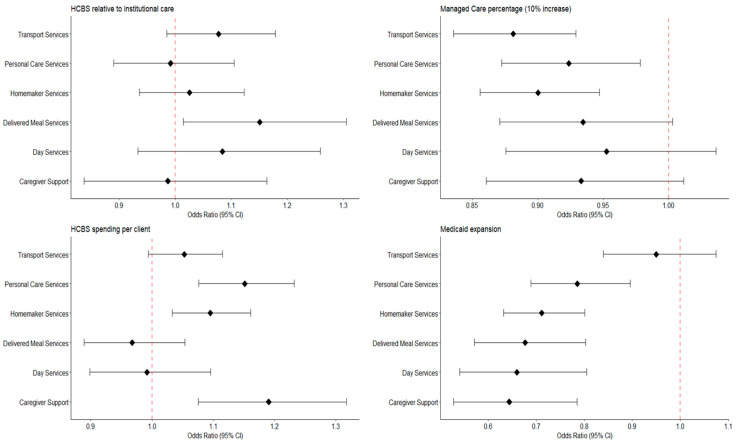
Associations of State-Level Factors With Service-Specific Unmet Service Needs Among Older Adult Consumers of Publicly- Funded Home- and Community- Based Services: The National Core Indicators- Aging and Disability Survey (2016–2019).

These findings were similar for several individual service categories, but with varying magnitude (Figure 2, Supplemental Table S2).

In analyses accounting for state-level clustering in the GEE model, confidence intervals became wide and very imprecise with *p* > 0.05 (Table 4, Supplemental Table S3). However, at the individual consumer level, funding through Managed LTSS was associated with significantly lower odds of consumers reporting unmet service needs even after accounting for state-level clustering (OR, 0.67; 95% CI, 0.47, 0.96; *p* < 0.05). Similarly, PACE was associated with significantly lower odds of consumers reporting unmet service needs even after accounting for state-level clustering (OR, 0.39; 95% CI, 0.27, 0.57; *p* < 0.001).

## 4. Discussion

In our study, using a large sample of HCBS consumers across 13 states, we found that Medicaid expansion and a higher percentage Medicaid beneficiaries enrolled in managed care were associated with lower odds of unmet service needs for HCBS consumers. Conversely, higher HCBS spending relative to institutional care was associated with greater odds of unmet service needs. The results show that there are meaningful differences across states in relation to HCBS consumers’ unmet service needs. After accounting for clustering of responses by state (13 states), estimates were imprecise with very wide confidence intervals. These findings suggest that the variability within states is key to account for, when examining the role of state factors on quality outcomes and drawing meaningful conclusions about the role of state-level factors on HCBS quality. This hypothesis should be validated in future studies. Consistent with previously published research, we found that the odds of unmet HCBS needs were significantly greater among consumers living with dementia, physical disability, and serious mental illness [14]. These findings also align with previous research suggesting poorer self-reported care experiences and quality of life among HCBS consumers living with dementia, physical disability, or serious mental illness [16].

Notably, we found that at the consumer level, Managed LTSS or PACE beneficiaries had significantly lower odds of unmet HCBS needs in fully adjusted models accounting for state-level clustering. The direction of this association was similar to that of state-level proportion of Medicaid beneficiaries enrolled in managed care on unmet HCBS needs. This result adds to a growing, though mixed, body of literature examining the impact of Managed LTSS and PACE on consumer outcomes [17,18,19]. Several prior studies have documented potential benefits of Managed LTSS and PACE, including improved care coordination, expanded access to HCBS, and reductions in institutional care use [18,19,20]. Evaluations in states like Virginia, Iowa, Tennessee, and New Jersey have reported that Managed LTSS implementation was associated with greater use of community-based services and higher satisfaction among enrollees [19,20,21,22]. However, other studies have noted variability in outcomes, with some stakeholders raising concerns about care plan implementation, network adequacy, and transparency [23]. Our findings contribute important evidence suggesting that, when appropriately monitored, Managed LTSS and PACE may help reduce gaps in care and better align service delivery with consumer needs [18,19,20]. These results underscore the value of state-level investment in managed care and coordinated care infrastructure that prioritizes person-centered planning, strong oversight, and robust accountability mechanisms to ensure equitable and responsive HCBS access [24]. Of note, enrollment in Managed LTSS may be subject to selection effects, as states vary in whether managed LTSS is mandatory or voluntary and in which populations are included, raising the possibility that observed differences in unmet HCBS needs could partly reflect differences in who is enrolled rather than the program model alone. We also highlight that substantial state-level variation in managed LTSS design including differences in covered services, care coordination requirements, and oversight mechanisms may contribute to heterogeneity in our results and should be explored in future research.

The unexpected finding that higher HCBS spending relative to institutional care was associated with greater unmet HCBS needs suggests that fiscal rebalancing may not automatically translate into improved service access [25]. One potential explanation which may be evaluated in future research is a “demand eruption effect,” in which greater investment and program visibility increase awareness and demand among consumers, outpacing the capacity of provider networks. At the same time, the complexity of service offerings and involvement of multiple agencies can create coordination challenges and gaps in quality oversight [25]. These dynamics may result in mismatches between policy priorities at the state level and on-the-ground implementation, with workforce shortages, limited provider capacity, and insufficient service management further constraining the ability of systems to meet consumer needs [25]. Together, these factors highlight the importance of pairing financial investments with deliberate efforts to strengthen care coordination, supply capacity, and quality monitoring. Higher unmet HCBS needs among states with higher spending HCBS may reflect deinstitutionalization requirements under Olmstead, where states must support people in the community if at all possible, and some of these people have very serious illnesses and disabilities. The association between Medicaid expansion and lower unmet HCBS needs may reflect broader access to coverage among low-income and vulnerable populations; however, the true effect of expansion likely varies by state [1,4]. While some states implemented expansion in ways that facilitated streamlined enrollment and comprehensive outreach, others adopted more restrictive approaches, such as limited outreach, burdensome administrative processes, or delayed implementation, which may have constrained the extent of benefit to HCBS users [26,27,28]. These differences indicate that the impact of Medicaid expansion on unmet HCBS needs is not uniform but instead depends on how states operationalize policy changes and connect newly eligible individuals to services [26]. From a policy perspective, ensuring that Medicaid expansion reaches its full potential for reducing unmet HCBS needs will require states to adopt simplified enrollment procedures, invest in outreach and navigation supports, and integrate eligibility expansions with HCBS delivery systems.

Our study is the first to examine how state-level factors influence consumer-reported unmet service needs in HCBS. Previous research has evaluated the impact of state-level factors on other measures of HCBS quality and performance. For instance, Segelman (2017) found that higher HCBS spending delayed nursing home admissions [7], while Wang (2021) showed that service breadth improved community discharge rates, but service intensity did not [8,9]. McGarry and Grabowski (2023) and Cheng (2024) reported that expanding HCBS reduced institutional care use and improved state LTSS performance [10,28]. Muramatsu and Campbell (2002) observed that greater HCBS spending increased formal assistance but not informal care for consumers [29], while Muramatsu (2010) linked HCBS support to lower depression rates in older adults [30]. It is important to note that most of these studies did not account for clustering by state. In our model that accounted for clustering of respondents within states, the estimates were very imprecise, i.e., had very wide confidence intervals.

Notably, while the effect sizes for many state-level variables remained reasonably large in the models that accounted for state clustering, they were not statistically significant. Our findings highlight the importance of adjusting for clustering by state to account for correlation among respondents within a state. This suggests that it may be important to avoid direct (unadjusted) comparisons of consumer-reported unmet service needs across states, as such comparisons may be misleading. This is especially critical when employing consumer-reported unmet service needs as a standardized measure of HCBS quality within national efforts such as value-based purchasing initiatives [31,32]. More research is needed to explore state-level variations in HCBS outcomes and to include additional variables beyond those in our current models [33,34].

Although our study uses data from 2016 to 2019, prior to the COVID-19 pandemic, the insights are directly relevant to the challenges HCBS systems now face. The pandemic disrupted HCBS delivery through workforce shortages, service suspensions, and increased reliance on informal caregivers, all of which may have magnified unmet HCBS needs [34]. At the same time, federal policies such as enhanced HCBS funding through the American Rescue Plan Act (ARPA) and new CMS requirements for quality reporting created opportunities for states to strengthen HCBS infrastructure [11,34]. Our findings therefore provide an important baseline against which to measure post-pandemic changes in unmet HCBS needs. States can use these results to better align financing and administrative structures with person-centered outcomes. For example, monitoring unmet service needs alongside spending ratios could help ensure that investments in HCBS rebalancing translate into actual service adequacy for consumers. Likewise, as managed care and PACE programs expand, states should incorporate consumer-reported outcomes into oversight to safeguard against potential service limitations. Finally, Medicaid expansion remains a critical lever for promoting equitable HCBS access, suggesting that coverage policy decisions continue to have downstream implications for unmet need. Next steps should include evaluating post-2020 data to understand whether pandemic-era policy changes, workforce interventions, and federal funding initiatives have reduced or exacerbated unmet HCBS needs. Comparative analyses of states that adopted different approaches to ARPA funding or telehealth integration, for instance, could potentially reveal strategies most effective in improving access and should be considered in future research.

This study has limitations. Data which come from administrative records may be prone to coding errors and the consumer-reported data in the NCI-AD survey could be influenced by recall bias. In our analyses accounting for clustering by state, we were limited by a very small number of clusters (13 states), which may bias our findings. Variability in state involvement between survey years may also affect our estimates and the generalizability of our findings. While the NCI-AD survey provides standard criteria for sample sizes and margins of error, states tailor their samples to address their specific concerns, which can differ widely in terms of eligibility, service structures, and demographics; further limiting generalizability. Even though we measured state-level factors in the year preceding the NCI-AD survey wave, the analyses are cross-sectional and observational which precludes from making causal inferences and may be susceptible to reverse causation. Additionally, even though our models account for key confounding variables, unmeasured and residual confounding might still influence the results.

## 5. Conclusions

In this multi-state analysis of NCI-AD data, we found that consumer-reported unmet HCBS needs were shaped by state-level factors, including Medicaid expansion, HCBS spending patterns, and managed care penetration, as well as program participation in managed LTSS and PACE. These findings underscore the importance of administrative and policy choices in determining consumers’ day-to-day access to needed services.

Looking ahead, these results suggest several implications for policy and practice. First, states seeking to reduce unmet HCBS needs should evaluate how financing priorities such as balancing HCBS versus institutional spending translate into actual consumer access. Second, the association between managed care and lower unmet HCBS needs highlights the importance of ensuring that managed LTSS and PACE models are designed and monitored to maintain person-centered quality. Third, Medicaid expansion continues to play a role in improving equity of access by broadening eligibility for vulnerable populations.

It is important to recognize that these data predate the COVID-19 pandemic and recent federal investments in HCBS. The pandemic exposed and exacerbated vulnerabilities in LTSS systems, including workforce shortages and disruptions in service delivery, while ARPA and other federal initiatives provided unprecedented new resources to states. Future research should build on our findings by assessing whether pandemic-era flexibilities (e.g., telehealth, payment adjustments, caregiver supports) and federal funding streams have altered patterns of unmet need. Understanding these shifts is essential for guiding sustainable reforms in HCBS financing, workforce development, and consumer protections. As publicly funded LTSS programs continue to evolve, linking administrative structures to consumer experiences offers a path forward for ensuring that HCBS truly meet the needs and preferences of older adults and people with disabilities.

## Figures and Tables

**Table 1:** Summary of Different Funding Programs Enabling Access to Home- and Community-Based Services in the United States of America.

Feature	Program of All-Inclusive Care for the Elderly (PACE)	Older Americans Act (OAA)	Managed Long-Term Services and Supports (Managed LTSS)	Medicaid Waiver LTSS (e.g., HCBS Waivers)
**Program Type**	A comprehensive, all-in-one healthcare program that combines medical and long-term care services into a single coordinated system. Example: InnovAge and Johns Hopkins ElderPlus.	A framework for discretionary grants that provides funding to states and communities for a wide range of social services for older adults. Example: Title III nutrition and caregiver programs managed by Area Agencies on Aging (AAAs).	A Medicaid-funded program in which states have a contract with managed care organizations (MCOs) to deliver long-term services and supports through a capitated payment system. Example: Arizona Long Term Care System (ALTCS).	A Medicaid option that allows states to waive certain federal requirements (e.g., institutional care mandates) in order to provide long-term services and support in home- and community-based settings. Example: New York’s OPWDD 1915(c) waiver for individuals with developmental disabilities.
**Eligibility**	Specific and strict. Participants must be at least 55 years old, certified by their state as needing a nursing home level of care, and able to live safely in the community at the time of enrollment. Example: PACE enrollment criteria require nursing-home level certification.	Broad. Services are generally available to people aged 60 and older, with a focus on those with the greatest economic or social need. Example: Meals on Wheels prioritizes homebound seniors with limited income or support.	Varies by state, but typically available to Medicaid-eligible individuals who need nursing home level of care and enroll in a managed care plan. Example: Florida Managed Medical Assistance LTC program serves Medicaid beneficiaries needing institutional-level care.	Varies by waiver and state. Typically limited to individuals who meet Medicaid income requirements and have a nursing home level of care need, but who can be served safely in the community. Example: California’s HCBS waiver for adults with disabilities and frail elders.
**Funding**	A managed care plan funded by fixed capitated payments from Medicare and Medicaid. The PACE provider receives a set amount per participant and is responsible for all their health and long-term care needs. Example: Dual-eligible PACE participants receive joint Medicare–Medicaid funding.	Funded through discretionary appropriations from Congress. The money is distributed to states and AAAs, which contract with local service providers. Example: Title III-B funds support transportation services through community nonprofits.	States receive federal Medicaid matching funds and pay MCOs a capitated rate to manage and deliver LTSS. Example: Texas STAR + PLUS uses capitated payments to MCOs for LTSS delivery.	Jointly funded by state and federal Medicaid dollars through Section 1915(c) or other waivers. Example: Wisconsin Family Care waiver funded with blended state and federal Medicaid dollars.
**Service Delivery**	An interdisciplinary team coordinates and provides all necessary medical and social services through a single PACE center. Services include adult day care, primary care, home care, and transportation. Example: PACE day health centers integrating clinic, therapy, and meals.	Community-based programs. Services are delivered by a network of providers, typically managed by AAAs, and are not necessarily integrated under a single entity. Example: Congregate meal programs run through senior centers.	Services are delivered through contracted provider networks managed by MCOs. Care coordination is typically required, but integration varies by state and plan. Example: MCO care coordinators in New Jersey’s Managed LTSS oversee both medical and support services.	Services are delivered through approved community-based providers. States have flexibility in designing benefits and provider networks, but services are not necessarily integrated. Example: Home health agencies delivering waiver-funded personal assistance.
**Scope of Services**	All-inclusive. Covers a full continuum of medical and support services, including hospital care, prescriptions, and long-term care, to keep participants independent for as long as possible. Example: PACE covers hospitalizations, medications, and LTSS under one plan.	Provides grants for specific programs, such as congregate and home-delivered meals, transportation, and caregiver support. It does not cover a participant’s full medical care like PACE does. Example: Title III-C funding supports Meals on Wheels programs.	Broader than OAA, narrower than PACE. Includes LTSS such as personal care, home modifications, and sometimes integrated medical care, depending on the state. Example: Minnesota’s MLTSS programs integrate acute and LTSS under MCOs.	Focused on LTSS, particularly home- and community-based services such as personal assistance, respite care, habilitation, adult day health, and case management. Does not usually cover all medical services. Example: Colorado’s HCBS waiver funds respite and habilitation services but not hospital care.
**Focus**	Keeping frail, high-need older adults who would otherwise require a nursing home in their community. A strong emphasis is placed on preventive care to reduce hospitalizations. Example: PACE prevents nursing home placement for dual-eligibles.	Supporting the health, independence, and well-being of older adults through a variety of social services. Example: OAA-funded transportation helps older adults maintain independence.	Promoting cost-effective care delivery and better outcomes by shifting LTSS into managed care arrangements, with a focus on integration, efficiency, and quality oversight. Example: Tennessee’s CHOICES program emphasizes cost savings and care integration.	Expanding access to community-based alternatives to institutional care, with emphasis on flexibility, consumer choice, and supporting individuals in the least restrictive setting possible. Example: HCBS waivers reduce nursing home use by funding in-home supports.

**Table 2:** Descriptive Characteristics of Study Population: The National Core Indicators- Aging and Disability Survey (2016–2019).

Characteristic	N = 13,654 ^1^
***State-Level Factors***	
HCBS spending relative to institutional care spending (ratio)	1.24 (0.90, 2.03)
Percentage of Medicaid beneficiaries in managed care	0.84 (0.80, 0.95)
Average HCBS spending per client	$18,500 ($12,600, $28,300)
Medicaid expansion	7758 (57%)
***Consumer-Level Factors***	
Age (years; median [IQR])	77 (71, 84)
Female (vs. not Female)	9791 (72%)
ADRD diagnosis	2059 (17%)
Physical Disability	6995 (58%)
Developmental Disability	1088 (9%)
Brain Injury	1455 (12%)
Mental Health Condition	2634 (20%)
Multiple Chronic Conditions	3500 (26%)
Funding Program	
Medicaid-A&D Waiver	4008 (29%)
Managed LTSS	4507 (33%)
PACE	621 (5%)
OAA	3386 (25%)
Other	1132 (8%)
Medicare Enrollee	11,093 (91%)
Have Legal Guardian	1141 (10%)
Marital Status	
Single	1567 (13%)
Married/Domestic Partner	2570 (21%)
Separated/Divorced	3053 (25%)
Widowed	4985 (41%)
ZIP Code RUCA Classification	
Metropolitan	9669 (72%)
Micropolitan	2065 (15%)
Rural	658 (5%)
Small town	1085 (8%)
Living Arrangement	
Alone	6569 (53%)
Family	5305 (43%)
Other	602 (5%)
Race/Ethnicity	
White	7931 (60%)
Black or African-American	3025 (23%)
Hispanic or Latino	1347 (10%)
Other/Multiracial/Multiethnic	930 (7%)
Overall Health	
Poor	2501 (19%)
Fair	5236 (39%)
Good	4075 (30%)
Very Good	1344 (10%)
Excellent	334 (2%)
Proxy	1780 (13%)
Service Use	
Personal Care Services	6491 (48%)
Homemaker Services	2961 (22%)
Delivered Meal Services	4157 (30%)
Day Services	1051 (7.7%)
Transport Services	1075 (7.9%)
Caregiver Support	757 (5.5%)

^1^ n (%) or Median (interquartile interval); IQR—interquartile interval; ADRD—Alzheimer’s Disease and Related Dementias; Medicaid-A&D waiver—Medicaid Aged & Disabled waiver; LTSS—Long term care supports and services; PACE—Program of All-Inclusive Care for the Elderly; OAA—Older Americans Act; HCBS—Home- and Community—Based Services.

**Table 3:** Associations of State-Level Factors With Overall Unmet Service Needs Among Older-Adult Consumers of Publicly Funded Home- and Community- Based Services: The National Core Indicators- Aging and Disability Survey (2016–2019).

Characteristic	Logistic Model
	Adjusted Odds Ratio (95% CI)
***State-Level Factors***	
HCBS spending relative to institutional care spending	1.19 (1.11, 1.28) ***
Percentage of Medicaid beneficiaries in managed care (per 10% increase)	0.92 (0.89, 0.96) ***
HCBS spending per client	1.00 (0.96, 1.05)
Medicaid expansion	0.80 (0.73, 0.87) ***
***Consumer-Level Factors***	
Funding Program	
Medicaid-A&D waiver	referent
Managed LTSS	0.67 (0.61, 0.74) ***
OAA	1.11 (1.00, 1.24)
PACE	0.39 (0.31, 0.49) ***
Other	0.91 (0.78, 1.06)
Female (vs. not Female)	1.00 (0.92, 1.09)
Marital Status	
Single	referent
Married/Domestic Partner	0.97 (0.83, 1.13)
Separated/Divorced	1.21 (1.06, 1.38) **
Widowed	0.99 (0.87, 1.13)
ZIP Code RUCA Classification	
Metropolitan	referent
Micropolitan	0.80 (0.71, 0.89) ***
Rural	0.73 (0.61, 0.88) **
Small town	0.87 (0.75, 1.00)
Living Arrangement	
Alone	referent
Family	1.03 (0.93, 1.14)
Other	0.77 (0.64, 0.94) **
Race/Ethnicity	
White	referent
Black or African-American	1.06 (0.96, 1.18)
Hispanic or Latino	1.15 (1.00, 1.33)
Other/Multiracial/Multiethnic	1.36 (1.16, 1.59) ***
Overall Health	
Poor	referent
Fair	0.67 (0.61, 0.75) ***
Good	0.48 (0.43, 0.54) ***
Very Good	0.40 (0.34, 0.46) ***
Excellent	0.32 (0.24, 0.43) ***
Medicare Enrollee (yes vs. no)	1.40 (1.21, 1.62) ***
Have Legal Guardian (yes vs. no)	0.90 (0.76, 1.05)
ADRD diagnosis (yes vs. no)	1.13 (1.01, 1.28) *
Physical Disability (yes vs. no)	1.12 (1.03, 1.22) **
Developmental Disability (yes vs. no)	0.95 (0.79, 1.15)
Brain Injury (yes vs. no)	1.16 (0.99, 1.37)
Mental Health Condition (yes vs. no)	1.39 (1.27, 1.53) ***
Proxy (yes vs. no)	0.99 (0.87, 1.12)

* *p* < 0.05; ** *p* < 0.01; *** *p* < 0.001; CI = Confidence Interval; ADRD—Alzheimer’s Disease and Related Dementias; Medicaid-A&D waiver—Medicaid Aged & Disabled waiver; LTSS—Long term services and supports; PACE—Program of All-Inclusive Care for the Elderly; OAA—Older Americans Act; HCBS—Home- and Community- Based Services.

**Table 4:** Logistic Regression with Clustered Standard Errors for Associations of State-Level and Administrative Factors With Overall Unmet Service Needs Among Older-Adult Consumers of Publicly Funded Home- and Community- Based Services: The National Core Indicators- Aging and Disability Survey (2016–2019).

Characteristic	GEE Model
	Adjusted Odds Ratio (95% CI)
***State-level factors***	
HCBS relative to institutional care	1.19 (0.85, 1.69)
Percentage of Medicaid beneficiaries in managed care (10% increase)	0.92 (0.79, 1.08)
HCBS spending per client	1.00 (0.81, 1.23)
Medicaid expansion	0.80 (0.54, 1.19)
***Administrative Factor- Funding Program***	
Medicaid-A&D waiver	referent
Managed LTSS	0.67 (0.47, 0.96) *
OAA	1.11 (0.72, 1.70)
PACE	0.39 (0.27, 0.57) ***
Other Programs	0.91 (0.61, 1.36)

* *p* < 0.05; *** *p* < 0.001. CI—Confidence Interval. GEE—Generalized estimating equation for logistic regression model, accounting for correlation between respondents from the same state. HCBS—Home- and Community- Based Services; LTSS—Long-term services and supports; Medicaid-A&D—Medicaid Aged & Disabled waiver; OAA—Older American’s Act; PACE—Program of All-Inclusive Care for the Elderly. Models are adjusted for all covariates in Table 1 with random effect for survey year-state cluster.

## Data Availability

Restrictions apply to the availability of these data. Data were obtained from the Health Services Research Institute (HSRI) and can be requested from them. The data use agreement between the University of Minnesota and the Health Services Research Institute (HSRI) does not allow sharing of NCI-AD data with the public.

## Supplementary Materials

The following supporting information can be downloaded at: https://www.mdpi.com/article/10.3390/jmahp13040051/s1, Table S1: Survey Questions for Service Use and Unmet Needs from National Core Indicators—Aging and Disability Survey; Table S2: Associations of State-Level Factors With Service Specific Unmet Needs Among Older-Adult Consumers of Six Individual Publicly-Funded Home- and Community- Based Services: The National Core Indicators- Aging and Disability Survey (2016–2019); Table S3: Generalized Estimating Equation (GEE) Model for Adjusted odds ratios (AOR) and 95% confidence intervals (CIs) for unmet need for all. Models are also adjusted for survey year.
