# Supplementary material for: Examining State Policies and Administrative Factors as Determinants of Consumer-Reported Unmet Service Needs in Publicly Funded Home- and Community-Based Services in the United States
Source: J Mark Access Health Policy. 2025 Oct 2;13(4):51. doi: 10.3390/jmahp13040051 (PMC12551106; doi:10.3390/jmahp13040051)
Supplement: Supplementary file 1 [file jmahp-13-00051-s001.zip › jmahp-3748914-supplementary.pdf]

Supplementary

Supplemental Methods

Survey methods

States voluntarily participate in the NCI-AD survey, employing a probabilistic sampling method to select participants from the eligible population for their services. The goal is to achieve a maximum margin of error of 5% with a 95% confidence level. Prior to conducting interviews, detailed background information on survey participants is systematically collected from administrative records, covering demographics, personal attributes, legal status, services utilized, and clinical diagnoses. This data is then merged with survey responses to create comprehensive profiles for each participant. Information from the survey's Background Information section, including race, gender, mental health diagnoses, Alzheimer's disease and related dementias, and disabilities, is typically obtained from state administrative channels such as case management, managed care, and Medicaid billing records. Administrative records are crucial for determining service types, often sourced from the Medicaid Management Information System.

Usually, sample selection is started within 3-4 months of annual survey wave initiation, with individuals potentially surveyed for up to 8 months following selection due to the extended data collection timeline. The NCI-AD background information section relies on state administrative records for demographic and service-related data. In cases where relevant administrative records are unavailable, individuals may be directly queried at the survey's conclusion. However, certain subsections of the Background Information section, such as primary long-term services and supports (LTSS) funding source, primary program, services received through the program, duration of participation, self-directed support, and legal guardianship status, require data retrieval from administrative records. To qualify for the survey, individuals must currently be receiving at least one active service (e.g., personal care, homemaker, transportation) at least twice a week for approximately 3 months, as active service receipt is a prerequisite for survey participation.

Supplemental Table S1. Survey Questions for Service Use and Unmet Needs from National Core Indicators – Aging and Disability Survey.

BI-16. What type of paid long-term care supports is the person receiving? [Check all that apply]	Non-HCBS Services:<br><br>__1 Skilled nursing facility/nursing home services<br><br>HCBS Services:<br><br>__2 Round-the-clock services (bundled services by an HCBS provider that has round-the-clock responsibility for the health and welfare of the person – may be provided in a person's home or in a congregate setting such as group home, shared living, assisted living, etc.).<br><br>__3 Home-based services (services that a person receives in his or her home or apartment, when the provider does not have round-the-clock responsibility for the person's health and welfare).<br><br>__3a Personal care services such as attendant care, personal care assistance (assistance with activities of daily living (ADLs), instrumental activities of daily living (IADLs) and/or health-related tasks, NOT including services required to be provided by a licensed home health agency or under the supervision of a licensed nurse or therapist).<br><br>__3b Homemaker services, chore services (performance of light or heavy housekeeping tasks, NOT including assistance with ADLs or other health-related tasks).

	__3c Companion services (supervision and/or social support, NOT including assistance with ADLs or other health-related tasks, or habilitation; may include performance of light housekeeping tasks).<br><br>__3d Other personal care services such as home health aide, etc.<br><br>__4 Day services (services other than supported employment usually provided outside of a person's home during the working day).<br><br>__4a Social adult day services (support services, NOT including health services or habilitation, provided to adults in a fixed site facility during the working day)<br><br>__4b Community integration services (services specifically intended to assist in participating in community activities, NOT including health services or habilitation; may include supports related to community participation that are provided in the person's residence)<br><br>__4c Adult day health<br><br>__4d Other day services such as prevocational services, day habilitation, education services, etc.<br><br>__5 Equipment, medical supplies, technology and modifications such as personal emergency response system<br><br>(PERS), home and/or vehicle accessibility adaptations, assistive technology, specialized medical equipment disposable medical

	supplies (purchase or rent of material items, devices, or product systems to improve or maintain a person's functional status).<br><br>__6 Nursing services such as private duty nursing, skilled nursing.<br><br>__7 Other health and therapeutic services not identified above such as health monitoring, medication management, occupational or physical therapy (services to support people in improving or maintaining health or functional capacity).<br><br>__8 Other mental health and behavioral services not identified above such as crisis intervention, behavior support, peer specialist, counseling, etc. (services to support people in improving or maintaining mental or behavioral health).<br><br>__9 Non-medical transportation not provided as part of another category such as round-the-clock services or day services. May include transportation to and from other waiver services, transportation to community activities, and/or the purchase of public transit tokens or passes.<br><br>__10 Case management<br><br>__11 Home delivered meals (prepared meals sent to a person's home).<br><br>__12 Employment assistance, supported employment such as job development, ongoing individual or group supported employment,

	career planning (assistance to help obtain or maintain paid employment or self-employment).<br><br>__13 Caregiver support such as respite and caregiver counseling and/or training (assistance to people who provide ongoing support to the service recipient, when assisting that support person is the primary purpose)<br><br>__14 Other services not listed (FILL IN)<br>____________________________________<br><br>__50 NONE<br><br>__98 Don’t know

In-Person Survey-83. Do the long-term care services you receive meet your current needs and goals?	_N/A – Person said they are not receiving services<br><br>_Yes, completely, all needs and goals<br><br>_Some needs and goals<br><br>_No, not at all, needs or goals are not met<br><br>_Don’t Know<br><br>_Unclear/refused/no response

In-Person Survey-84. What additional long-term care services might help you meet your needs and goals? [Check all that apply	_N/A – Services meet all needs<br>_Skilled nursing facility, nursing home services<br>_Personal care assistance, personal care services<br>_Home maker/chore services<br>_Healthcare home services, home health<br>_Home delivered meals<br>_Adult day services<br>_Transportation<br>_Respite/family caregiver support<br>_Health care<br>_Mental health care<br>_Dental care<br>_Housing assistance<br>_Heating/cooling assistance<br>_Hospice<br>_Funeral Planning

	_Other [fill in]<br><br>_Don’t Know<br><br>_Unclear/refused/no response

Supplemental Table S2. Associations of State-Level Factors With Service Specific Unmet Needs Among Older-Adult Consumers of Six Individual Publicly-Funded Home- and Community- Based Services: The National Core Indicators- Aging and Disability Survey (2016-2019).

	Personal Care	Homemaker/ Chore	Delivered Meals	Adult Day services	Transportatio n	Health Care	Caregiver Support
**Characteristic**	**AOR (95% CI)**[12]	**AOR (95% CI)**[12]	**AOR (95% CI)**[12]	**AOR (95% CI)**[12]	**AOR (95% CI)**[12]	**AOR (95% CI)**[12]	**AOR (95% CI)**[12]
Actual service	1.01 (0.92, 1.12)						
HCBS relative to institutional care	1.25 (0.83, 1.89)	1.94 (0.97, 3.88)	1.30 (0.88, 1.92)	1.14 (0.74, 1.75)	1.68 (0.98, 2.87)	1.02 (0.71, 1.45)	1.47 (1.03, 2.11)*
Managed care percentage (10% increase)	1.31 (1.13, 1.52)***	1.74 (1.52, 1.99)***	1.20 (1.04, 1.39)*	1.16 (0.98, 1.38)	1.30 (1.14, 1.49)***	0.87 (0.74, 1.01)	0.92 (0.79, 1.08)
HCBS spending per client	1.04 (0.76, 1.43)	0.92 (0.56, 1.51)	1.11 (0.83, 1.49)	1.16 (0.83, 1.62)	0.90 (0.62, 1.32)	1.34 (1.00, 1.79)*	0.94 (0.70, 1.26)
Medicaid expansion	0.65 (0.36, 1.18)	0.38 (0.14, 1.02)	0.56 (0.31, 1.03)	0.67 (0.33, 1.35)	0.70 (0.34, 1.47)	0.83 (0.47, 1.48)	0.68 (0.38, 1.22)
Program							
MEDICAID	—	—	—	—	—	—	—
MLTSS	0.91 (0.79, 1.05)	1.10 (0.96, 1.26)	1.17 (0.98, 1.41)	1.05 (0.84, 1.30)	1.01 (0.89, 1.15)	0.70 (0.56, 0.87)**	0.92 (0.71, 1.18)

NF	1.46 (0.69, 3.11)	1.19 (0.53, 2.63)	1.19 (0.43, 3.31)		0.76 (0.32, 1.80)	1.33 (0.45, 3.95)	1.05 (0.25, 4.39)
PACE/OAA/Other	1.04 (0.91, 1.20)	1.77 (1.58, 2.00)***	1.24 (1.05, 1.47)*	1.30 (1.09, 1.56)**	1.29 (1.15, 1.45)***	0.86 (0.72, 1.04)	1.38 (1.11, 1.70)**
Female (vs not Female)	1.13 (1.03, 1.24)**	1.08 (0.99, 1.17)	1.08 (0.96, 1.21)	1.03 (0.91, 1.17)	1.13 (1.04, 1.23)**	0.99 (0.87, 1.13)	0.94 (0.81, 1.10)
Marital Status							
Single	—	—	—	—	—	—	—
Married/Domestic Partner	0.92 (0.81, 1.06)	1.09 (0.96, 1.24)	1.13 (0.94, 1.36)	0.96 (0.79, 1.17)	0.69 (0.61, 0.79)***	1.11 (0.92, 1.34)	0.99 (0.79, 1.24)
Separated/Divorced	0.96 (0.85, 1.09)	1.02 (0.90, 1.15)	1.15 (0.99, 1.35)	1.09 (0.92, 1.30)	0.88 (0.79, 0.99)*	0.89 (0.72, 1.08)	0.93 (0.76, 1.14)
Widowed	0.97 (0.85, 1.12)	0.94 (0.83, 1.08)	1.11 (0.93, 1.33)	0.94 (0.76, 1.15)	0.78 (0.69, 0.89)***	1.16 (0.94, 1.44)	0.71 (0.55, 0.92)**
ZIP Code RUCA Classification							
Metropolitan	—	—	—	—	—	—	—
Micropolitan	0.80 (0.70, 0.90)***	0.86 (0.77, 0.97)*	1.00 (0.85, 1.17)	0.73 (0.60, 0.89)**	0.84 (0.75, 0.95)**	1.03 (0.86, 1.24)	0.92 (0.74, 1.14)
Rural	0.91 (0.74, 1.12)	0.99 (0.83, 1.19)	1.06 (0.81, 1.38)	0.76 (0.56, 1.05)	0.85 (0.70, 1.03)	0.98 (0.74, 1.31)	1.00 (0.71, 1.42)

Small town	0.74 (0.63, 0.88)***	0.85 (0.73, 0.98)*	0.86 (0.70, 1.06)	0.70 (0.55, 0.90)**	0.84 (0.73, 0.97)*	0.99 (0.79, 1.23)	0.83 (0.63, 1.10)
Living Arrangement							
Alone	—	—	—	—	—	—	—
Family	1.05 (0.95, 1.17)	0.90 (0.82, 0.99)*	0.90 (0.79, 1.02)	1.0 (0.86, 1.15)	0.84 (0.77, 0.93)***	3.00 (2.53, 3.55)***	1.03 (0.87, 1.23)
Other	0.50 (0.41, 0.61)***	0.41 (0.34, 0.50)***	0.53 (0.41, 0.68)***	0.60 (0.47, 0.76)***	0.64 (0.54, 0.76)***	0.38 (0.26, 0.54)***	0.85 (0.64, 1.13)
Race/Ethnicity							
White	—	—	—	—	—	—	—
Black or African-American	1.10 (0.99, 1.23)	0.88 (0.79, 0.98)*	1.16 (1.01, 1.33)*	1.23 (1.05, 1.44)**	0.96 (0.87, 1.06)	1.00 (0.84, 1.17)	1.00 (0.82, 1.21)
Hispanic or Latino	1.22 (1.00, 1.49)	0.98 (0.80, 1.20)	0.88 (0.68, 1.14)	0.90 (0.66, 1.23)	0.95 (0.79, 1.16)	0.99 (0.75, 1.31)	1.13 (0.80, 1.58)
Other/Multiracial/Multiethnic	1.32 (1.13, 1.53)***	1.24 (1.07, 1.44)**	1.18 (0.96, 1.44)	1.36 (1.10, 1.66)**	1.24 (1.07, 1.44)**	1.13 (0.93, 1.38)	1.16 (0.89, 1.50)
Overall Health							
Poor	—	—	—	—	—	—	—
Fair	0.70 (0.63, 0.78)***	0.76 (0.69, 0.84)***	0.81 (0.71, 0.92)**	0.93 (0.79, 1.09)	0.74 (0.67, 0.82)***	0.79 (0.67, 0.92)**	0.74 (0.63, 0.88)***

Good	0.49 (0.43, 0.55)***	0.57 (0.51, 0.63)***	0.61 (0.52, 0.71)***	0.91 (0.77, 1.09)	0.58 (0.52, 0.65)***	0.78 (0.66, 0.93)**	0.47 (0.38, 0.58)***
Very Good	0.37 (0.31, 0.44)***	0.45 (0.38, 0.53)***	0.51 (0.40, 0.63)***	0.78 (0.62, 0.99)*	0.47 (0.40, 0.55)***	0.76 (0.60, 0.95)*	0.44 (0.32, 0.59)***
Excellent	0.31 (0.23, 0.43)***	0.36 (0.27, 0.47)***	0.29 (0.18, 0.46)***	1.10 (0.80, 1.50)	0.35 (0.27, 0.46)***	0.73 (0.53, 1.01)	0.38 (0.23, 0.62)***
Medicare Enrollee	0.96 (0.86, 1.06)	0.88 (0.79, 0.98)*	0.99 (0.86, 1.14)	0.85 (0.74, 0.99)*	0.95 (0.86, 1.06)	0.95 (0.82, 1.11)	0.94 (0.79, 1.12)
Have Legal Guardian	0.95 (0.80, 1.14)	0.92 (0.77, 1.10)	0.75 (0.59, 0.96)*	1.30 (1.04, 1.63)*	0.96 (0.81, 1.13)	1.11 (0.89, 1.38)	0.95 (0.70, 1.29)
ADRD	1.15 (1.00, 1.32)*	0.98 (0.85, 1.12)	1.02 (0.85, 1.24)	1.22 (1.00, 1.49)*	0.77 (0.66, 0.89)***	1.42 (1.19, 1.70)***	0.72 (0.55, 0.95)*
Physical Disability	1.25 (1.12, 1.39)***	1.20 (1.08, 1.32)***	0.95 (0.84, 1.08)	0.97 (0.84, 1.13)	1.13 (1.02, 1.24)*	1.07 (0.92, 1.25)	1.24 (1.03, 1.49)*
Developmental Disability	1.23 (1.05, 1.44)*	1.03 (0.88, 1.20)	1.09 (0.89, 1.35)	1.66 (1.35, 2.04)***	1.21 (1.04, 1.40)*	1.34 (1.09, 1.65)**	1.23 (0.95, 1.59)
Brain Injury	1.06 (0.92, 1.22)	0.95 (0.83, 1.09)	1.06 (0.89, 1.26)	1.08 (0.90, 1.30)	1.09 (0.96, 1.24)	1.22 (1.01, 1.48)*	1.11 (0.89, 1.38)
Mental Health Condition	1.23 (1.12, 1.35)***	1.31 (1.20, 1.43)***	1.41 (1.26, 1.59)***	1.35 (1.19, 1.55)***	1.40 (1.28, 1.52)***	1.13 (0.98, 1.29)	1.34 (1.14, 1.56)***
Age group							
Over65	—	—	—	—	—	—	—

Under65	1.02 (0.91, 1.14)	0.89 (0.80, 0.99)*	1.27 (1.11, 1.46)***	1.33 (1.13, 1.56)***	1.05 (0.95, 1.17)	1.16 (0.98, 1.37)	1.22 (1.01, 1.47)*
Proxy	1.29 (1.13, 1.47)***	0.82 (0.71, 0.94)**	0.80 (0.65, 0.97)*	1.10 (0.92, 1.33)	0.68 (0.59, 0.79)***	2.47 (2.12, 2.88)***	1.01 (0.78, 1.29)
Actual service	1.01 (0.92, 1.12)						
Actual service		0.86 (0.77, 0.95)**					
Actual service			0.83 (0.71, 0.96)*				
Actual service				1.09 (0.86, 1.38)			
Actual service					0.94 (0.82, 1.07)		
Actual service						1.15 (0.93, 1.43)	
Actual service							0.97 (0.71, 1.31)

[1]*p<0.05; **p<0.01; ***p<0.001

[2]CI = Confidence Interval

Supplemental Table S3. Generalized Estimating Equation (GEE) Model for Adjusted odds ratios (AOR) and 95% confidence intervals (CIs) for unmet need for all . Models are also adjusted for survey year.

	GEE Logistic Model
Characteristic	AOR (95% CI)[1,2]
HCBS relative to institutional care	1.16 (0.82, 1.63)
Managed care percentage (10% increase)	0.91 (0.77, 1.07)
HCBS spending per client	1.01 (0.83, 1.23)
Medicaid expansion	0.80 (0.54, 1.21)
Program	
MEDICAID	—
MLTSS	0.70 (0.49, 1.00)*
PACEOAAOTH	0.93 (0.67, 1.29)
Female (vs not Female)	0.99 (0.91, 1.08)
Marital Status	
Single	—
Married/Domestic Partner	1.00 (0.86, 1.15)
Separated/Divorced	1.23 (1.08, 1.41)**
Widowed	1.02 (0.91, 1.15)

ZIP Code RUCA Classification	
Metropolitan	—
Micropolitan	0.82 (0.67, 1.01)
Rural	0.76 (0.54, 1.08)
Small town	0.90 (0.65, 1.26)
Living Arrangement	
Alone	—
Family	1.03 (0.92, 1.14)
Other	0.75 (0.57, 0.99)*
Race/Ethnicity	
White	—
Black or African-American	1.07 (0.88, 1.29)
Hispanic or Latino	1.05 (0.67, 1.65)
Other/Multiracial/Multiethnic	1.33 (1.00, 1.79)
Overall Health	
Poor	—
Fair	0.67 (0.60, 0.75)***
Good	0.49 (0.43, 0.54)***

Very Good	0.39 (0.34, 0.46)***
Excellent	0.32 (0.22, 0.46)***
Medicare Enrollee	1.34 (1.05, 1.72)*
Have Legal Guardian	0.90 (0.74, 1.09)
ADRD	1.09 (0.95, 1.25)
Physical Disability	1.15 (0.95, 1.39)
Developmental Disability	0.97 (0.74, 1.28)
Brain Injury	1.14 (0.95, 1.38)
Mental Health Condition	1.39 (1.24, 1.57)***
Proxy	1.02 (0.87, 1.21)

[1]*p<0.05; **p<0.01; ***p<0.001

[2]CI = Confidence Interval
